# Clinical use of statins in hematopoietic stem cell transplantation: Old drugs and new horizons

**Published:** 2016-01-01

**Authors:** Mehdi Mohammadi, Mohammad Vaezi, Bahador Mirrahimi, Molouk Hadjibabaie

**Affiliations:** 1 Clinical Pharmacy Department, Faculty of Pharmacy, Tehran University of Medical Sciences, Tehran, Iran; 2 Hematology-Oncology and Stem Cell Research Center, Tehran University of Medical Sciences, Tehran, Iran; 3 Clinical Pharmacy Department, Faculty of Pharmacy, Shahid Beheshti University of Medical Sciences, Tehran, Iran; 4 Research Center for Rational Use of Drugs, Tehran University of Medical Sciences, Tehran, Iran

**Keywords:** Infection, GvHD, Statin, Stem cell transplantation

## Abstract

Hydroxymethylglutaryl Co-enzyme A reductase inhibitors, also known as statins, are a class of anti-hyperlipidemic agents. These drugs have been employed vastly to reduce the morbidity and mortality of cardiovascular disorders. Soon after their introduction, benefits other than their primary actions were discovered. Along with these pleiotropic properties, a series of mainly favorable effects has been proposed in patients intended to undergo hematopoietic stem cell transplantation. These actions address some complications encountered by this special population such as graft-versus-host disease, efficacy of chemotherapy, infections, etc. This review presents the current evidence surrounding these issues.

## Introduction

 Inhibitors of 3-hydroxy3-methylglutaryl Coenzyme A reductase or statins were developed primarily to reduce the risk of cardiovascular diseases. They were used later to address several new indications unrelated to the primary lipid-lowering effect. Among them, new promising roles have been proposed in hematopoietic stem cell transplantation (HSCT).^   1 ^

Although HSCT is a life-saving approach for various types of hematological malignancies, it imposes noticeable treatment-related morbidity and mortality on patients. ^2^^-^^4^  A number of recently published studies have evaluated the possible advantageous roles of statins in some complications encountered after HSCT. Our focus has been more on Graft vs. Host Disease (GvHD), infection risk, efficacy of chemotherapy, and hypercholesterolemia. Here, we will scrutinize these emerging proceedings.

**Statins and the risk of GvHD**

GvHD is one of the main challenges of HSCT survivors. Both animal and human studies implicating various statins have demonstrated the effectiveness of these agents in this setting; 5^,^^6^  but depending on whether the drug had been administered to the donor or the recipient, inconsistent results were obtained. Rotta et al. evaluated transplant outcomes of 1206 patients undergoing allogeneic HSCT retrospectively.^        5 ^ Donors were either HLA-identical siblings or HLA-matched unrelated ones. Recipients with a history of statin use for at least 3 months prior to transplantation were considered as “on statin treatment”. Results were adjusted for age, gender, donor type, conditioning intensity, source of stem cells, and specific calcineurin inhibitor (CNI) agent used for GvHD prophylaxis. There were no differences in grade II-IV acute GvHD (aGvHD), non-relapse mortality and overall mortality between statin users and non-users. However, less extensive chronic GvHD (cGvHD) occurred in recipients previously treated with statin. The latter effect was observed in patients treated with cyclosporine only and not in those who received tacrolimus as GvHD prophylaxis. This effect also was associated with more frequent recurrence of underlying malignancy. The same authors evaluated the effect of donor statin use on the incidence of GvHD in another retrospective study.^        7 ^

Five hundred and sixty-seven patients were included. After adjustment for other variables in multivariable analysis, donor statin use was associated with reduced rate of grade III-IV but not grade II-IV aGvHD. Again, this effect was observed only in patients who were prescribed cyclosporine as post-engraftment prophylaxis. There was no difference between the two groups in terms of cGvHD. After further examination of involved organs, more protective effects were observed in the gastrointestinal tract involvement and not in the skin. Similar results have been obtained in a trial conducted by Hamadani et al.

In this phase II study, simultaneous donor and recipient pretreatment with atorvastatin 40 mg daily orally for 14-28 days before cell harvest was well tolerated and resulted in reduced incidence of acute GvHD.^        8 ^ Of note, donors in this study were matched siblings and recipients were treated with tacrolimus as the immunosuppressant agent of choice. In general and based on limited evidence available, it can be hypothesized that donor statin treatment is associated with reduced incidence of aGvHD, while recipient pretreatment with statin may cause less severe cGvHD. More prospective randomized trials are needed to further scrutinize these preliminary results.

Besides HSCT, immunosuppressive therapy is needed in solid organ transplantation and some autoimmune disorders. Statins also seem promising as new add-on therapies in these states which will be discussed later in this article.

**Statins and the risk of infection in post-transplantation period**

There is not sufficient evidence available about the effects of these drugs on the risk of infections after HSCT. One study used the same database as one of the articles mentioned above.^   9 ^ In this retrospective analysis, neither the donor nor the recipient’s statin use was found to reduce the risk of some infections. These included CMV infection, bacteremia, invasive fungal infections and lower respiratory tract infections. Those recipients who were taking statins; however, experienced more cumulative episodes of gram-negative bacteremia that did not result in increased mortality. Donor’s statin use was also associated with increased viral respiratory tract infections.

Due to the retrospective design of this study and also due to the lack of prospective trials, it is very difficult to conclude any beneficial or detrimental effect of statin use on the risk of infections in this population.

**Statin use to enhance chemotherapy efficacy in potential transplantation candidates**

Proliferation of multiple myeloma (MM) cells is inhibited in the presence of statins. It has been hypothesized that statin exposure leads in release of some caspases (namely caspase 9, 3, and 8) with subsequent apoptosis of MM cells.^        10 ^ Not all MM cell lines are sensitive to statins.^   11 ^ In preliminary clinical studies, these drugs appeared to be promising anti-myeloma agents. In a phase II pilot study, simultaneous simvastatin could prevent drug resistance in MM cases resistant to two cycles of bortezomib or bendamustine.^        12 ^ Enrolled patients were six post-autologous HSCT cases who received two cycles of bortezomib or bendamustine with no response.

In this case, the patients received two additional cycles plus simvastatin 80 mg daily. Simvastatin was administered from day -2 to two days after completing chemotherapy. Results were compared with another group that included 10 patients with resistant MM after 4 cycles of bortezomib or bendamustine, but without concomitant simvastatin. In 3 out of 6 patients, serum level of M protein was increasing even after two cycles of chemotherapy, but when simvastatin was added to the ongoing regimen, protein levels markedly declined. In two other patients, stable serum M protein levels began to fall consistent with the initiation of simvastatin. In only 1 patient, protein level was not affected by simvastatin treatment. In this patient; however, serum level of cholesterol increased slightly in a paradoxical manner that indicated insufficient HMG-CoA reductase inhibition. The only important limitation of this study was the small number of samples.

In another phase II trial, contradictory results were obtained with simvastatin in relapsed or refractory multiple myeloma patients.^        13 ^ Simvastatin 15 mg/kg/day divided as twice daily was added to chemotherapy regimen in days 1-7 of each cycle. Twenty-eight day cycles consisted of vincristine 0.4 mg, doxorubicin 9 mg/m^2^ and dexamethasone 40 mg orally on day 7-10. Response evaluation was performed after two cycles of treatment and in the case of stability; treatment was continued for two more cycles.

Disease progression after two cycles was the factor that led to discontinuation of treatment. Interim analysis was performed after recruitment of 12 patients. Initial assessment showed that one patient achieved a partial response, 6 patients were stable and other 5 patients progressed. Five out of 6 patients with stable disease were those with progressive disease before enrollment. Stability was maintained for 94-258 days (with a median of 103 days). According to the authors’ opinion, this rate of response was not noticeable enough to justify completion of the study and it was discontinued prematurely.

In a different survey, the effect of statin use at the time of autologous HSCT on transplantation outcomes was explored.^        10 ^ Hamadani et al. retrospectively analyzed the records of 146 patients undergoing high dose chemotherapy with melphalan (200 mg/m^2^) followed by autologous HSCT for multiple myeloma. Statin use was defined as taking any statin agent with doses ≥ 20 mg per day from 1 month before to 1 month after transplantation. Twenty-eight statin-users were compared with 118 patients who did not take statin. Complete response (CR) and very good partial response (VGPR) did not differ significantly between groups (43% vs. 45%, p=0.84), but the composite endpoint of overall response rate (CR + VGPR + PR) trended more favorable with statin use versus non-use (97% vs. 78%, p=0.07). Median overall survival (25.7 vs. 22 months, p=0.65) and progression-free survival (19.5 vs. 14.8 months, p=0.97) were not significantly different between groups. Based on the available body of evidence, it is not clear whether co-administration of statin with chemotherapy will result in enhanced efficacy or not. Well-designed randomized trials with large sample sizes are needed to better investigate this capacity.

**Statin use to treat hypercholesterolemia after transplantation**

With improving survival of post-HSCT patients; however, concerns have been raised about long-term non-relapse mortality. Cardiovascular disorders comprise a major constituent of a large variety of long-term complications. ^14^^-^^17^  In a retrospective analysis carried out by Kagoya et al. risk factors, prevalence, and prognosis of hypercholesterolemia in patients after allogeneic HSCT were examined.^   18 ^

Medical records of 194 patients who survived more than 100 days after transplantation came into the analysis. Hypercholesterolemia (defined as cholesterol levels above 240 mg/dL in at least 2 consecutive measurements one week apart) occurred in 83 of 194 patients (42.8%). Hypertriglyceridemia (defined as triglyceride levels beyond 200 mg/dL in at least two occasions one week apart) developed in 99 patients (50.8%). These abnormalities led to the administration of statins to 19, fibrate to 2, combined statin-fibrate to one, and nicotinic acid to one patient. Non-relapse mortality did not differ significantly between patients with or without hypercholesterolemia (6-year mortality 17.8% vs. 18.7% respectively; p=0.83).

In contrast to some other studies,^19^^,^^20^ use of CNIs was not an independent risk factor for either hypercholesterolemia or hypertriglyceridemia. Nonetheless, both cGVHD and subsequent corticosteroid use were associated with hypercholesterolemia. Statins have been previously used to treat lipid abnormalities in solid organ transplantations. ^21^^,^^22^  Lipid abnormalities have been successfully treated with statins. In those who did not receive cholesterol-lowering therapy, normalization of values occurred shortly after HSCT, signifying the impact of timely approach to cGvHD on lipid profile values. Another unexpected finding in the study was the lower rate of relapse of primary disease in patients with hypercholesterolemia. Nonetheless, this finding can be attributed to liver involvement due to cGvHD and ensuing graft versus leukemia effect.

In one of the largest studies conducted to date, Blaster et al. followed 1493 patients who underwent allogeneic HSCT and survived more than 100 days after transplantation.^        23 ^ Of whom, 732 patients never had done tests to measure cholesterol level and were subsequently excluded from the study. Both hypercholesterolemia and hypertriglyceridemia were defined as any single outpatient value of ≥ 200 mg/dL.

All patients with a prescription for statin for at least 30 days after the procedure were considered as statin user. Ninety-five percent of patients received tacrolimus as the CNI of choice for GvHD prophylaxis. Sirolimus was prescribed for 50% of patients. The cumulative incidence of aGvHD and cGvHD was 26% and 60% at 2 years post-transplant, respectively. Out of the 761 patients, 556 had at least one cholesterol level equals to or greater than 200 mg/dL and were considered hypercholesterolemic according to the National Cholesterol Education Panel ATP-III guideline. Out of the 761 patients, 560 had at least one lipid value measurement before transplantation which revealed 32.0% (n=179) prevalence of dyslipidemia prior to transplantation. A high proportion of patients also suffered from post-transplantation hypertriglyceridemia (n=531, 72.5%). Among patients with both pre- and post-transplant triglyceride values, the mean peak values were 171 and 275 mg/dL, respectively (a mean alteration of 109 mg/dL, p< 0.0001). In terms of the impact of transplantation procedure on lipid values, 249 of 381 patients (65%) with both pre- and post-procedural measurements, developed newly-diagnosed abnormal lipid values after transplantation. Given this rate of de novo conversion, it is noteworthy to say that 164 of 179 previously hypercholesterolemic patients remained unchanged after procedure. Patients with pre-existing hypercholesterolemia had significantly higher cholesterol levels compared with de novo cases (259 mg/dL vs. 275 mg/dL, p=0.004 respectively). Similar results were also observed in triglyceride status. Approximately, 64% of patients with pre-transplant TG level <200 mg/dL developed elevated TG levels after the procedure. Again, pre-existing hypertriglyceridemic patients exerted higher TG levels after transplantation compared with de novo subjects (398 vs. 305 mg/dL, p< 0.001). Suffering from higher grades of aGvHD was associated with higher risk for abnormal cholesterol levels (frequency of 81% for aGvHD grade II-IV vs. 71% for aGvHD grade 0-I, p=0.007). These abnormal results of lipid values led to the prescription of statins to 220 of 761 patients (29%) within 2 years after transplantation. Adverse drug reactions attributed to statin were negligible in only one case after drug discontinuation. Results of other trials unrelated to the HSCT population have also reported new favorable effects. In all of these situations, statins have been used as an adjunct to immunosuppressive regimens and have improved some indices of morbidity or mortality.

Statins have prolonged the function of allografts and improved survival in cardiac transplant patients. ^24^^-^^27^  Beneficial effects in lung transplantation^        28 ^ and some autoimmune disorders such as rheumatoid arthritis and multiple sclerosis have also been reported. ^29^^-^^33^  Together, these data suggest that statin use after HSCT not only can normalize lipid values but also may enhance immune tolerance.

**Future look at statins in HSCT**

Regarding the positive effects of statins after myocardial infarction, hopes for new indications of these drugs have increased. At least a part of these positive effects has been attributed to the mobilization of endothelial progenitor cells from bone marrow. ^34^^-^^36^  Whether similar effects on hematopoietic progenitor cells may really exist remains to be determined in future trials, but a retrospective trial showed some positive results.^   37 ^ In this study, 86 patients with MM were retrospectively analyzed. All patients received G-CSF for stem cell mobilization. The outcome of leukapheresis of 20 patients on statin treatment was compared with the other 66 non-users. The chance of adequate cell harvest with first leukapheresis was marginally superior in statin users (85% vs. 63.6%, p=0.07). This may be the subject of future trials since poor mobilizations is a barrier to successful transplantation in autologous HSCT.^   38 ^ A summary of evidence reviewed in this article is shown in Table 1.

**Barriers to use of statins in post-HSCT population**

Despite the aforementioned benefits, some concerns may prevent widespread use of statins. Among them, potentials for adverse drug reactions and drug interactions appear to be the most bothersome. The two major adverse effects attributed to statins are liver injury and myopathy. Liver injury is best described as an idiosyncratic reaction that is neither predictable nor dose-dependent. True incidence remains to be clarified since surrogate markers are lacking^   39 ^ and diagnosis has been traditionally based on different scoring tools that stratify the reaction based on some clinical and laboratory data. ^40^^,^^41^  In the case of hepatotoxicity, different approaches do exist in the literature^42^^,^^43^ that underscores the need for more practical tools to establish the causality. Regardless of whether this toxicity really exists or not, the true prevalence remains low. This fear should not obscure the benefits of the drug even in patients with pre-existing liver disease.^44^^,^^45^ 

Muscle toxicity is another precaution for statin use. Various definitions have been proposed by accredited authorities. ^46^^-^^48^  Unlike liver toxicity, this effect occurs in a dose-dependent manner. Accumulating evidence suggest that reduction in metabolites produced by mevalonate pathway is the main mechanism involved in muscle toxicity.^   49 ^

Another concern in clinical practice is the potential of these drugs to interact with some medications used by HSCT patients. Most statins are substrates of P-glycoprotein (P-gp). This protein acts as an efflux pump which drops out the drugs into the gut lumen and reduces their absorption. Through the action of transporters such as organic anion-transporting polypeptide 1B1 (OATP1B1), more statin is picked up by the liver and available for metabolism by CYP enzymes. Drugs like cyclosporine interact with several steps of statin metabolism, and cause an elevation in serum level with subsequent risk of myopathy.^   50 ^ Such interactions have also resulted in clinical cases of rhabdomyolysis. ^51^^,^^52^  Each agent of this class exhibits a unique metabolic pathway with different potential for drug-drug interactions. Detailed review of these pharmacokinetic properties is beyond the scope of this article and is discussed elsewhere. ^50^^,^^53^  In general, the well-defined risk profile of these agents has failed to prevent their widespread use for their primary indications. It seems that it is also true in the case of HSCT population.

## CONCLUSION

 Besides traditional lipid-lowering potential, statins have shown promising benefits in HSCT. Their primary action of controlling cholesterol level is extremely efficient because of the high prevalence of lipid disorders reported by abovementioned trials. Depending on donor or recipient statin use, varying outcomes have been observed regarding GvHD. Infections do not seem to be favorably affected by the action of these drugs. Although not thoroughly investigated, a positive effect of statins is anticipated on the efficacy of conditioning regimens prior to autologous HSCT or pre-transplantation chemotherapy of MM. The stem cell mobilizing potential needs more investigation. Nevertheless, whether these effects are agent-specific for certain statins have not been investigated. Other than their classic effect, unfortunately these new roles have been surveyed either in retrospective studies or in small-size prospective trials. More well-designed prospective trials are needed to establish new indications for these drugs.

**Table 1:** Summary of studies on the use of statins in HSCT

**Study**	**Design**	**Year**	**Number of patient**	**Comparison**	**Main results**	**Comments**
**GvHD**						
Rotta et al.	Retrospective	2010	1206	Recipients with statin use vs. non-users	Less extensive cGvHD in statin users	No difference in grade II-IV aGvHD, Positive results only in patients taking cyclosporine
Rotta et al.	Retrospective	2010	567	Donor with statin use vs. non-use	Less grade III-IV aGvHD	No difference in cGvHD, Positive effects only in patients taking cyclosporine
Hamadani et al.	Prospective	2013	30	Atorvastatin use by both donor and recipient vs. none	Less aGvHD	Positive effects in patient on tacrolimus treatment
**Infection**						
Seo et al.	Retrospective	2013	1206	Recipient or donor use of statin vs. non-use	No appreciable effect on the risk of infections	Increased risk of gram-negative bacteremia with recipient statin use, Increased risk of respiratory tract viral infections with donor statin use
**Chemotherapy efficacy**						
Schmidmaier et al.	Prospective Pilot Phase II	2007	6	Simvastatin added to bendamustine or bortezomib vs. bendamustine or bortezomib alone	Serum M protein declined in almost all patients after addition of simvastatin	No significant adverse effect with addition of simvastatin
Van der Spek et al.	Prospective Phase II	2007	12	Simvastatin added to VAD vs. literature- based data for efficacy of VAD alone	No significant result at interim analysis	
Hamadani et al.	Retrospective	2008	146	Statin use at the time of autologous HSCT for MM vs. non-use	OR trended better with statin use (not significant)	CR and VGPR did not differ significantly, A trend toward increased stem cell mobilization with statin use
**Hyperlipidemia**						
Kagoya et al.	Retrospective	2012	194	-	Hypercholesterolemia in 42.8% and hypertriglyceridemia in 50.8% of patients	Successful treatment with statins
Blaster et al.	Retrospective	2012	1493	-	Dyslipidemia in 32.0% of patients prior to transplantation, Post-procedure elevated TG in 72.5% of patients	Only one statin discontinuation because of adverse effects with 220 prescriptions
**Stem cell mobilization**						
Stravodimou et al.	Retrospective	2014	86	Statin use vs. non-use in autologous HSCT for MM	A non-significant increase in stem cell mobilization	
